# Low Oxygen Storage Improves Tomato Postharvest Cold Tolerance, Especially for Tomatoes Cultivated with Far-Red LED Light

**DOI:** 10.3390/foods10081699

**Published:** 2021-07-22

**Authors:** Fahrizal Yusuf Affandi, Jan A. Verschoor, Maxence J. M. Paillart, Julian C. Verdonk, Ernst J. Woltering, Rob E. Schouten

**Affiliations:** 1 Horticulture and Product Physiology, Wageningen University and Research, P.O. Box 16, 6700 AA Wageningen, The Netherlands; julian.verdonk@wur.nl (J.C.V.); ernst.woltering@wur.nl (E.J.W.); Rob.schouten@wur.nl (R.E.S.); 2 Bioresource Technology and Veterinary Department, Vocational College, Universitas Gadjah Mada, Yogyakarta 55281, Indonesia; 3 Food & Biobased Research, Wageningen University and Research, P.O. Box 17, 6700 AA Wageningen, The Netherlands; jan.verschoor@wur.nl (J.A.V.); maxence.paillart@wur.nl (M.J.M.P.)

**Keywords:** chilling injury, controlled atmosphere, far-red

## Abstract

We investigated the effects of low oxygen storage on chilling injury development, colour development, respiration and H_2_O_2_ levels of ‘Merlice’ tomatoes cultivated with and without far red (FR) LED lighting during 20 days of shelf-life. Mature green (MG) and red (R) tomatoes were stored at 2 °C in combination with 0.5, 2.5, 5 and 21 kPa O_2_ for 15 days (experiment 1). MG tomatoes cultivated under either white LED or white LED light with FR LED light were stored at 2 °C in combination with 1, 5 and 21 O_2_ kPa for 14 days (experiment 2). Chilled MG and R tomatoes from experiment 1 showed decay, firmness loss and higher weight loss during shelf-life which were reduced under low oxygen conditions. FR during cultivation improved chilling tolerance of MG tomatoes. Fastest colour development and lowest respiration rate during shelf-life were observed for MG fruit cultivated with FR lighting prior to storage at 1 kPa O_2_/0 kPa CO_2_. H_2_O_2_ levels during the shelf-life were not affected during cold storage. The improved cold tolerance of MG tomatoes cultivated with FR lighting is likely due to lower oxygen uptake that led to both higher lycopene synthesis and less softening.

## 1. Introduction

Tomato (*Solanum lycopersicum*) is a chilling sensitive fruit that will develop a disorder called chilling injury (CI) when exposed to low, but above freezing temperatures [1]. Chilling stress disrupts metabolic processes and causes alterations in membrane fluidity, followed by an increase in reactive oxygen species (ROS) production. In addition, low enzymatic activity causes reduced ROS scavenging, which promotes development of CI symptoms [2,3,4]. CI symptoms in tomatoes include surface pitting, interrupted pigment (lycopene) synthesis, rapid softening, loss of aroma and production of off-flavours, as well as increased susceptibility to fungal infection [5,6]. CI symptoms usually become visible during a shelf-life period after fruits have been exposure to chilling temperatures [5,6,7].

Controlled atmosphere (CA) storage and Modified Atmosphere Packaging (MAP) have been shown to reduce CI in mango, Japanese plum, guava, avocado and persimmon [8,9,10,11,12,13]. Low oxygen reduces respiration rate, and in addition, it may decrease ethylene production and ethylene sensitivity. CA storage downregulated the expression of ACC-synthase and ACC-oxidase genes, responsible for ethylene synthesis [14]. It may also limit ROS production, which could alleviate chilling injury symptoms [10,15,16]. CA storage induced activation of antioxidant scavenger enzymes such as catalase (CAT), superoxide dismutase (SOD), ascorbate peroxidase (APX) and glutathione reductase (GR) in Japanese plum, apple and litchi [10,17,18], reducing ROS, often represented by lower hydrogen peroxide (H_2_O_2_) levels. H_2_O_2_ is both a toxic metabolite and signaling molecule [19,20]. Storage under CA slowed down the activities of cell wall degrading enzymes involved in lignification and softening [21,22]. In addition, low oxygen storage stabilised group VII of ethylene response factors (ERFVIIs) and transported these to the nucleus which induced expression of hypoxia-responsive genes. Hypoxia-responsive genes encode enzymes involved in sucrose catabolism (β-amylase, sucrose synthase and phosphofructokinase), fermentative metabolism (pyruvate decarboxylase, lactate dehydrogenase and alcohol dehydrogenase) and ROS scavenging (SOD, APX and CAT) [23,24,25].

The severity of CI symptoms depends on the ripening stage of the fruits; mature green (MG) tomatoes are more sensitive to CI than red (R) tomatoes [2]. Comparing the responses of R and MG fruit to chilling stress is expected to provide insights into the mechanism of how low oxygen alleviates CI in sensitive tomatoes [26,27,28]. We showed that addition of far-red (FR) lighting during cultivation alleviated CI in tomato. In MG fruit, additional FR lighting reduced weight loss, pitting and enhanced red colour development during shelf-life after prior cold storage. R fruit cultivated with additional far-red light were firmer at harvest and demonstrated reduced weight loss and less decay during shelf-life after prior cold storage [29]. In the current study we investigated the effect of varying low oxygen levels on CI occurrence in mature green (MG) and red (R) tomatoes during postharvest storage. In addition, we investigated the effect of FR lighting during cultivation on CI tolerance after prior low oxygen storage.

## 2. Materials and Methods

We carried out two experiments. In experiment 1, mature green (MG) and red (R) tomatoes were stored for 15 days at 2 °C either under regular atmosphere (21 kPa O_2_, RA) or under 0.5, 2.5 and 5 kPa O_2_, followed by a shelf-life period of 15 days at 20 °C. In experiment 2, MG tomatoes cultivated with or without FR were harvested and stored either under RA or under 1 and 5 kPa O_2_ followed by a shelf-life period of 15–20 days at 20 °C. In both experiments, decay index, colour and firmness, respiration rate and hydrogen peroxide (H_2_O_2_) level were determined at harvest, during cold storage and during subsequent shelf-life.

### 2.1. Plant Material and Growth Conditions

For the first experiment, mature green (MG) and red (R) ‘Merlice’ tomatoes were harvested from a commercial greenhouse in Bleiswijk, the Netherlands in November 2016. The colour stage of the fruit was assessed using the NAI index (see Section 2.5). MG tomatoes were defined as tomatoes with a NAI value between −0.77 and −0.6 at harvest. R tomatoes were defined as having NAI values between 0.25 and 0.55 at harvest. For the second experiment, MG ‘Merlice’ tomatoes were harvested from a greenhouse at Wageningen University in May 2019 of plants grown under white LED lighting (WL) or WL with 8.3 µmol m^−2^ s^−1^ FR lighting, with a peak at 730 nm. For the FR treatment, 6% of the photons in the red region were replaced with FR. This resulted in 13 µmol m^−2^ s^−1^ FR in the FR treatment and hence this treatment was called WL + 13FR and the photon flux density was kept constant at 215 µmol m^−2^ s^−1^. The greenhouse compartment was divided into four plots. The light intensity was 215 µmol m^−2^ s^−1^ at the top of the canopy. In this experiment, VYPRx PLUS modules (Fluence, TX, USA) were used as top lighting. For each of the plots there were six modules installed. Overhead lamps were switched on 16 h before sunset and switched off at sunset. Additionally, LED lighting was automatically switched off when the incoming sunlight exceeded 300 μmol m^−2^ s^−1^. The spectral composition of the light treatments is shown in Figure S1. Light treatments were separated by double sided, non-transparent, white reflective plastic sheets. At harvest, uniform MG fruits were selected with a NAI value between −0.77 and −0.6. Further greenhouse management (fertigation, pollination) was conducted according to standard commercial practice.

### 2.2. Experimental Setup

In experiment 1, MG and R tomatoes were randomly assigned into five tomatoes per maturity per CA treatment at harvest, at the end of CA storage for 15 days at 2 °C and during subsequent shelf-life at 5, 10 and 15 days. This amounts to 125 MG and 125 R tomatoes. At harvest, colour and firmness was measured for all tomatoes. At each sampling point, colour, firmness and CI indices measurements were carried out. In experiment 2, the effect of far red illumination at harvest was characterised by randomly selecting 40 MG tomatoes per light treatment. Eight tomatoes per light treatment per CA treatment were assigned as a replicate of four tomatoes for repeated non-destructive measurement at harvest, after 7 and 14 days of CA storage, and after 4, 7, 10, 14 and 21 days of subsequent shelf-life. Prior to sampling during at 7 days CA storage, the CA was stop and tomatoes were taken out to be analysed. Eight randomly assigned tomatoes per light treatment and per CA treatment were taken for destructive analysis at 7 and 14 days of CA storage and after 7, 14 and 21 days of shelf-life. In total 240 FR and 240 non-FR cultivated MG tomatoes were selected for this experiment.

Tomatoes were individually marked on three positions on the equator for repeated colour and firmness measurements during shelf-life. In addition, fresh weight and three chilling indices were assessed approximately every 3 days during shelf-life. Individual fruits, assigned for destructive measurements, were cut into small pieces and quickly frozen in liquid nitrogen and later ground into a fine powder for H_2_O_2_ measurements.

### 2.3. CA storage Conditions

Tomatoes were stored at 2 °C and 95% relative humidity (RH) under low oxygen conditions followed by subsequent shelf-life at 20 °C in darkness. Desired oxygen conditions were achieved by flushing humidified gas mixtures at a flow rate of 500 mL min^−1^ through 70 L stainless steel containers filled with tomatoes with an average weight of 5.15 ± 0.25 kg per container. In both experiments, tomatoes stored at RA and 2 °C served as low oxygen control whereas tomatoes stored at 12 °C and 95% RH under RA served as temperature control. All control treatment were carried out in identical containers and flow rate with the low oxygen treatments.

In experiment 1, MG and R tomatoes were subjected to low oxygen conditions of 0.5 kPa, 2.5 and 5 kPa O_2_ combined with 0 kPa CO_2_ (completed with balanced N_2_) for 15 days. Following cold storage, fruit were transferred to shelf-life conditions at 20 °C and 85% RH for 15 days. In experiment 2, MG tomatoes were subjected to low oxygen storage at 1 and 5 kPa O_2_ with 0 kPa CO_2_ (completed with balanced N_2_). During CA storage, respiration measurements were conducted. After 14 days of cold storage, tomatoes were exposed to shelf-life condition at 20 °C and 95% RH for 21 days.

### 2.4. Respiration Measurements

In experiment 2, respiration measurements were carried out according to method previously described by our group [30]. Analysis was carried out using an Interscience Compact GC system (Interscience, Breda, NL, USA) equipped with an RT-QBond column for detecting CO_2_ at the back channel and a MolSieve 5A coupled with a back pressure column type RT-QBond for the detection of O_2_ at the front channel. Helium with a constant pressure of 60 and 80 kPa was used as carrier gas for the back and front channel, respectively. Each column was connected to a Thermal Conductivity Detector (TCD) set at 110 °C. CGCeditor software (v1.5.5, 2008) was used to control the setting of the CompactGC. GC was continuously connected to the samples via tubing connected to a VICI valve (model EMTMA-CE). Valve and CompactGC were coordinated by EZChrom Elite software (v3.32 SP2).

Gas measurement were conducted directly from the container. Before measurement took place, the flow through the container was stopped to allow accumulation of CO_2_ and depletion of O_2_ and the first GC measurement was carried out. The second measurement was carried out at the end of the incubation period. The accumulation period was approximately 5 h. The difference in gas partial pressure between the first and second GC measurements was converted into consumption and production rates according to ideal gas law methods [31]. The measurement was carried out at day 4, 6, 10 and 12 during CA storage.

### 2.5. Colour and Firmness Measurement

Colour was assessed non-destructively by a hand-held photodiode array spectrophotometer (Pigment Analyzer PA1101, CP, Ibbenbüren, Germany). Remittance was assessed at 570 (R570) and 780 (R780) nm by calculating the normalised different vegetative index (*NDVI*, Equation (1)) and normalised anthocyanin index (*NAI*, Equation (2)) which are normalised value between −1 and 1 [32].

(1) $$NDVI=\frac{R_{780}-R_{660}}{R_{780}+R_{660}}$$

(2) $$NAI=\frac{R_{780}-R_{570}}{R_{780}+R_{570}}$$

Firmness was measured non-destructively using a commercial acoustic firmness tester (AFS, AWETA, Nootdorp, the Netherlands) with the tick power of the plunger set to 15. The AFS combines the single tomato resonant frequency (*f* in Hz) and mass (*m*, in kg), measured by an inbuild balance, into a *FI* (firmness index) [33] (Equation (3)).

(3) $$FI=\frac{f^{2}m^{2/3}}{10^{4}}$$

### 2.6. Disorder Index and Weight Loss

CI was assessed by three indices, a pitting index and uneven ripening for MG fruit, and a decay index for R tomatoes according to the previously described method [29]. All indices were visually assessed with the percentage of the tomato surface assigned to five classes (0 = no injury, 1 = <10%, 2 = 11–25%, 3 = 26–40%, 4 = >40% affected area). The average score of pitting and uneven ripening index for MG, and decay index tomatoes were termed general disorder index. Tomato weight loss over time was expressed as the percentage weight loss (*WL*, in %) with *W*_0_ the initial weight (in g) and *W_t_* the weight (in g) according to Equation (4).

(4) $$WL=\frac{W_{0}-W_{t}}{W_{0}}\times 100$$

### 2.7. Hydrogen Peroxide (H_2_O_2_) Measurement

H_2_O_2_ was quantified via a colorimetric method [34]. Briefly, a 300 mg sample of frozen and ground tissue per tomato was extracted in a solution containing of 0.75 mL 0.1% (*w*/*v*) trichloroacetic acid (TCA), 0.75 mL 10 mM phosphate buffer (pH 7) and 1.5 mL 1 M KI. The homogenate was centrifuged (15,000× *g*, 4 °C, 15 min) and the supernatant transferred to a new tube and allowed to sit at RT for 20 min before obtaining the absorbance at 390 nm using a Varian CARY 4000 spectrophotometer (Agilent, Santa Clara, CA, USA). Measured absorbances were converted into H_2_O_2_ concentrations using a calibration curve constructed with a commercial H_2_O_2_ solution (Sigma Aldrich, St. Louis, MO, USA).

### 2.8. Statistical Analysis

Data obtained during shelf-life were subjected to mixed ANOVA, applying SPSS ver.21 (SPSS, Chicago, IL, USA) at *p* < 0.05. Data from the first experiment were analysed by mixed ANOVA with oxygen level and maturity as between subject factors and days in storage as within subject factor. For the second experiment, mixed ANOVA was carried out with oxygen level and FR as between subject factor and days in storage as within subject factor. Normality of the variables was tested applying the Shapiro-Wilk test. Mauchly’s test of sphericity was carried out to test whether variances of the differences between all possible pairs of within-subject conditions were equal. If the sphericity assumption was not fulfilled, Greenhouse-Geisser’s correction was applied to calculate the degrees of freedom. In case of a significant interaction, a pairwise comparison was carried out for each shelf-life day with LSD (Least Significant Difference) values estimated.

## 3. Results

### 3.1. Experiment 1: Effects of Low Oxygen Conditions on CI Indices, Weight- and Firmness Loss

**Figure 1:**
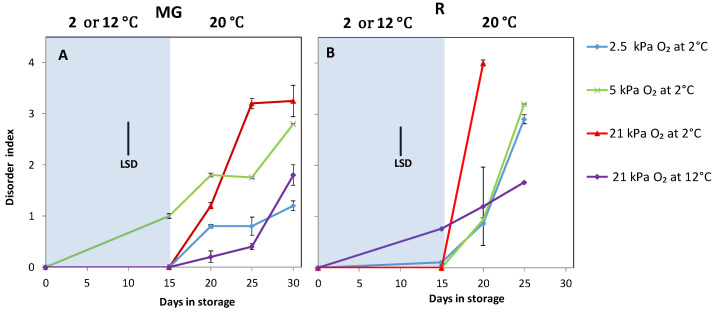
Chilling injury symptoms as indicated by the disorder index of MG (**A**) and R (**B**) tomatoes during cold storage at 2 or 12 °C (blue area) and subsequent shelf-life at 20 °C (white area). Blue, green, red and purple lines and symbols indicate 2.5, 5, 21 kPa O_2_ (low oxygen control) applied during storage at 2 °C and 21 kPa O_2_ at 12 °C (temperature control), respectively. The average decay index with indicated standard error is shown for five tomatoes. LSD values (*p* < 0.05) are indicated per panel. Disorder in MG fruit was determined by averaging the values of the pitting and uneven ripening index; disorder in R fruit was determined by the average decay incidence.

In the first experiment, typical CI symptoms such as pitting, uneven colouring and decay were observed for both MG and R tomatoes during low oxygen storage and shelf-life. Storage at 0.5 kPa oxygen resulted in necrosis, fungal infection and rotting and were therefore omitted from this study. In MG tomatoes there were generally no visible CI symptoms observed during cold storage, except for tomatoes stored at 5 kPa O_2_ (Figure 1A). During the shelf-life, fruit (MG and R), prior stored at 2.5 kPa O_2_, showed the lowest, and RA the highest disorder (Figure 1). MG tomatoes from the temperature control (12 °C) also developed some pitting, comparable to the tomatoes stored at 2.5 kPa O_2_. R tomatoes stored at 12 °C (temperature control) developed the least decay. At 2 °C, the R tomatoes stored at 2.5 kPa showed the least decay while the fruit stored at RA developed the highest disorder after 20 days of shelf-life which prevented further measurements. On the other hand, R tomatoes from the temperature control (21 kPa at 12 °C) developed the lowest decay (*p <* 0.0001). This indicated that the storage at 12 °C also resulted in a small amount of CI symptoms. In general, MG tomatoes developed slower pitting than R tomatoes, indicating that R tomatoes were surprisingly more sensitive to cold storage than MG tomatoes.

**Figure 2:**
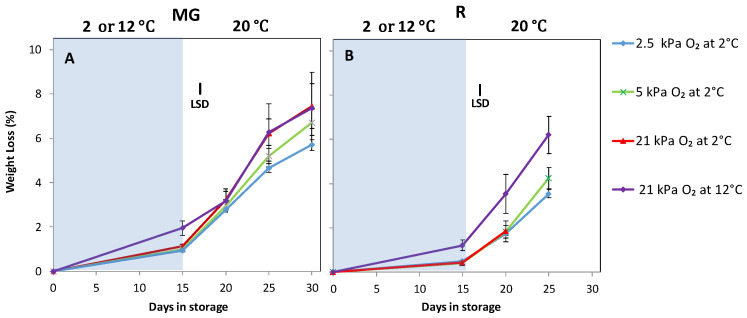
Weight loss of MG (**A**) and R (**B**) tomatoes during cold storage at 2 or 12 °C (blue area) and subsequent shelf-life at 20 °C (white area). Blue, green, red and purple lines and symbols indicate 2.5, 5, 21 kPa O_2_ (low oxygen control) applied during storage at 2 °C and 21 kPa O_2_ at 12 °C (temperature control), respectively. The average weight loss with indicated standard error is shown for five tomatoes. LSD values (*p* < 0.05) are indicated per panel.

**Figure 3:**
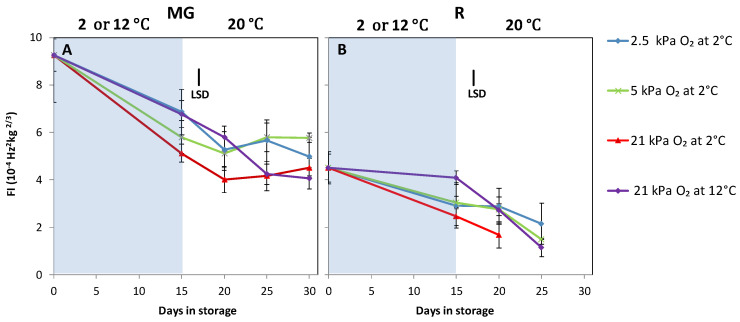
Firmness index of MG (**A**) and R (**B**) tomatoes during cold storage at 2 or 12 °C (blue area) and subsequent shelf-life at 20 °C (white area). Blue, green, red and purple lines and symbols indicate 2.5, 5, 21 kPa O_2_ (low oxygen control) applied during storage at 2 °C and 21 kPa O_2_ at 12 °C (temperature control), respectively. The average firmness index with indicated standard error is shown for five tomatoes. LSD values (*p* < 0.05) are indicated per panel.

Weight loss was higher for MG compared to R tomatoes (Figure 2). Fruit stored at 12 °C showed highest weight loss. The lowest weight loss for both MG and R tomatoes was observed in fruit that had been stored at 2 °C and 2.5 kPa O_2_ (*p <* 0.005). Fruit stored at 12 °C and stored at 2.5 or 5 kPa O_2_ at 2 °C showed less softening compared to fruit stored at 2 °C and 21 kPa O_2_ (Figure 3).

### 3.2. Experiment 1: Effects of Low Oxygen Conditions on Tomato Colour Development

**Figure 4:**
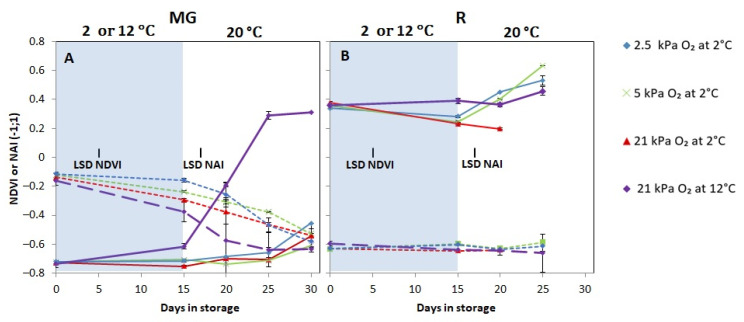
Colour as indicated by NDVI (dotted lines) and NAI (full lines) index of MG (**A**) and R (**B**) tomatoes during cold storage at 2 or 12 °C (blue area) and subsequent shelf-life at 20 °C (white area). Blue, green, red and purple lines and symbols indicate 2.5, 5, 21 kPa O_2_ (low oxygen control) applied during storage at 2 °C and 21 kPa O_2_ at 12 °C (temperature control), respectively. The NDVI or NAI with indicated standard error is shown for five individual tomatoes (repeated measure over times). LSD values (*p* < 0.05) are indicated per panel.

Red colour formation for MG fruit, as indicated by NAI values, was limited for all fruit that had been stored at 2 °C, independent of the oxygen level. Fruit stored at 12 °C showed colouration during subsequent shelf life at 20 °C. (Figure 4A). During low oxygen storage, more chlorophyll breakdown was observed with increasing oxygen levels. In R tomatoes, all treatments, except for the tomatoes in the temperature control, showed a reduction in the NAI values during cold storage. During shelf-life, fruit from all treatments showed increasing NAI values, except for the RA control (Figure 4B).

### 3.3. Experiment 2: Effects of Low Oxygen Storage of Mature Green Tomatoes Cultivated with and without Far Red Lighting on CI Symptoms, Weight- and Firmness Loss

**Figure 5:**
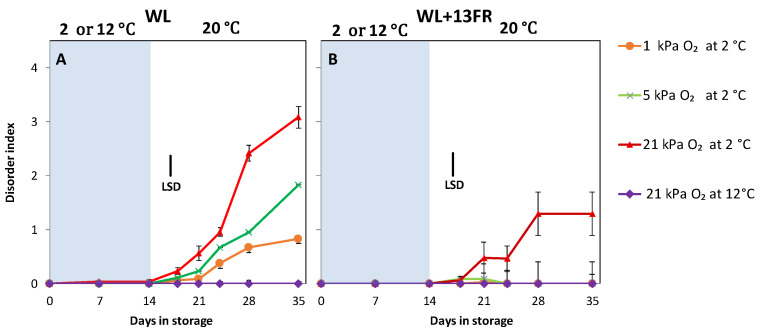
Chilling injury symptoms as indicated by the disorder index of MG tomatoes cultivated under white LED light (WL) without far red lighting (**A**) or with far red lighting during cultivation (**B**) during storage (blue area) at 2 or 12 °C and shelf-life at 20 °C (white area). Orange, green, red and purple lines and symbols indicate 1, 5 and 21 kPa O_2_ (low oxygen control) applied during storage at 2 °C, and 21 kPa O_2_ at 12 °C (temperature control), respectively. The average disorder index with indicated standard error is shown for two replicates of four tomatoes (*n* = 2); (repeated measure over times). LSD values (*p* < 0.05) are indicated per panel.

Tomatoes cultivated without far red lighting during cultivation showed CI symptoms during shelf-life. The lowest pitting index was observed for MG tomatoes stored at 1 kPa O_2_, the highest for the low oxygen control (Figure 5A). MG tomatoes cultivated with far-red lighting demonstrated reduced CI compared with tomatoes grown without FR lighting. In fact, no CI symptoms were observed for all low oxygen treatments, even after 3 weeks of shelf-life (Figure 5B). There were no chilling symptoms in fruit stored at 12 °C, and no differences were observed in terms of weight loss (Figure S2).

**Figure 6:**
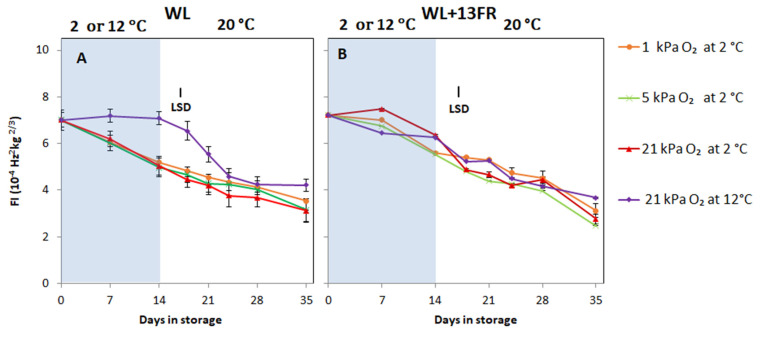
Firmness as indicated by firmness index (FI) of MG tomatoes cultivated under white LED light (WL) without far red lighting (**A**) or with far red lighting during cultivation (**B**) during cold storage (blue area) at 2 or 12 °C and subsequent shelf-life at 20 °C (white area). Orange, green, red and purple lines and symbols indicate 1, 5 and 21 kPa O_2_ (low oxygen control) applied during storage at 2 °C, and 21 kPa O_2_ at 12 °C (temperature control), respectively. The average firmness index with indicated standard error is shown for two replicates of four tomatoes (*n* = 2); (repeated measure over times). LSD values (*p*< 0.05) are indicated per panel.

Firmness at harvest was similar for MG tomatoes cultivated with or without FR lighting (*p* > 0.05). Softening during storage at 2 °C for was faster for MG tomatoes that were cultivated without- compared to those with FR lighting (*p*< 0.05) (Figure 6). Softening of tomatoes cultivated without FR was similar during storage and shelf-life independent of the storage oxygen concentration. Tomatoes cultivated without FR from the temperature control treatment showed no softening during storage (Figure 6A), but tomatoes cultivated with FR showed similar softening for all treatments (Figure 6B).

### 3.4. Effects of Low Oxygen Conditions on Colour Development of Mature Green Tomatoes Cultivated with and without Far Red Lighting

**Figure 7:**
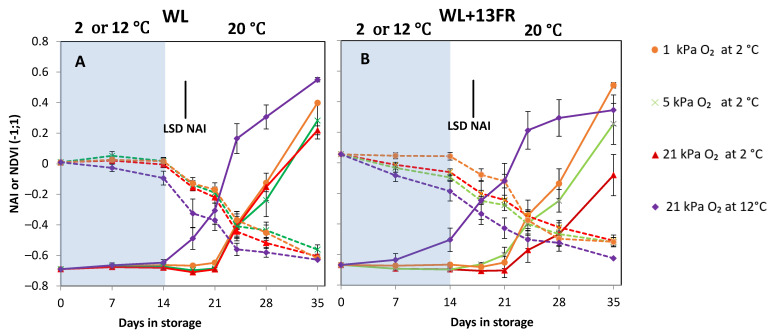
Colour indicated by NDVI (dotted lines) or NAI (full lines) values of MG tomatoes cultivated under white LED light (WL) without far red lighting (**A**) or with far red lighting during cultivation (**B**) during cold storage (blue area) at 2 or 12 °C and subsequent shelf-life at 20 °C (white area). Orange, green, red and purple lines and symbols indicate 1, 5 and 21 kPa O_2_ (low oxygen control) applied during storage at 2 °C, and 21 kPa O_2_ at 12 °C (temperature control), respectively. The average NDVI and NAI values with indicated standard error are shown for two replicates of four tomatoes (*n* = 2). LSD values (*p* < 0.05) are indicated per panel.

During cold storage red colour development was blocked, as indicated by the constant NAI values, irrespective of low oxygen treatments for both MG fruit cultivated with and without FR lighting. Colour development for the temperature control tomatoes started immediately, although faster for the MG tomatoes cultivated with FR lighting (Figure 7). During shelf-life, colour development was similar for the different low temperature oxygen storage treatments in fruit without FR lighting. Fruit cultivated with FR lighting reached higher NAI values in fruit prior stored at the low oxygen concentrations (*p <* 0.001) (Figure 7B). NDVI values were not significantly affected by oxygen level nor FR treatment.

### 3.5. Effects of Low Oxygen Conditions on Respiration and H_2_O_2_ Production of Mature Green Tomatoes Cultivated with and without Far Red Lighting

**Figure 8:**
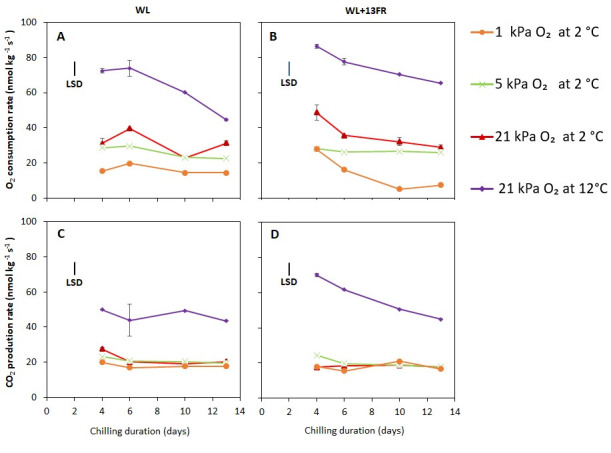
Respiration MG tomatoes cultivated under white LED light (WL) without- (**A**,**C**) or with far red lighting during cultivation (**B**,**D**) indicated as oxygen consumption (**A**,**B**) or CO_2_ production (**C**,**D**) measured during cold storage at 2 or 12 °C. Orange, green, red and purple lines and symbols indicate 1, 5 and 21 kPa O_2_ (low oxygen control), and 21 kPa O_2_ at 12 °C (temperature control), respectively. The average O_2_ or CO_2_ production rates with indicated standard error are shown for two replicates from each respective container (*n* = 2) during cold storage at 2 °C or 12 °C. LSD values (*p* < 0.05) are indicated per panel.

Respiration rate measurements were carried out from the fourth day onwards to allow time to achieve the set low oxygen conditions. The O_2_ consumption rate at 2 °C was observed to be lower for MG tomatoes stored at lower oxygen levels (Figure 8A,B). At 12 °C, both CO_2_ production and O_2_ consumption was higher than at 2 °C. The CO_2_ production rate, however, was similar at the low oxygen levels (Figure 8C,D). The oxygen consumption rate over time was lower for MG fruits cultivated with FR lighting and stored at 1 kPa O_2_.

H_2_O_2_ levels were stable during cold storage and steadily increased in all treatments during subsequent shelf-life (*p* < 0.0001, Figure S3). Varying oxygen levels during cold storage showed similar patterns of H_2_O_2_ production during subsequent shelf-life.

## 4. Discussion

### 4.1. Low Oxygen Storage Alleviated CI in Tomato Which Might Be Related to Lower Oxygen Uptake and Improved Lycopene Synthesis

When low temperature was combined with reduced oxygen concentrations, lower decay and lower weight loss was observed during shelf-life for both MG and R tomatoes (Figure 1 and Figure 2). Our results showed that O_2_ consumption decreased with lower oxygen levels while CO_2_ production rates were similar (Figure 8). Low oxygen storage is reported to suppress respiration and ethylene production [15,35]. Low oxygen uptake might reduce O_2_ availability for ROS production, such as singlet oxygen (^1^O_2_) and superoxide anions (O_2_^.−^) [36]. O_2_^−^ is dismutated into H_2_O_2_ by the action of SOD [4,36,37]. Lower levels of O_2_^−^ are expected to yield lower levels of H_2_O_2_. However, we did not observe a lower level of H_2_O_2_ in the low oxygen stored fruit (Figure S3), perhaps indicating that low oxygen did not suppress oxidative stress initiated by the presence of O_2_^−^. As tomato stored under low oxygen showed further red colouration close to or even higher than the non-chilled control (Figure 4B) and faster red colouration (Figure 7) after transfer to 20 °C, we hypothesise that lycopene acted directly to quench ^1^O_2_. Carotenoids are able to quench ^1^O_2_ due to its high number of conjugated double bonds, whereas lycopene and its precursors, are the most effective ^1^O_2_ quencher [38,39,40,41]. Quenching of ^1^O_2_ by lycopene or its precursors might have resulted in delayed lycopene synthesis or lycopene degradation [31,42]. Therefore, uninterrupted colour synthesis might indicate that low oxygen prevents lycopene degradation as well as preserving the lycopene biosynthetic machinery during cold storage allowing new lycopene synthesis during shelf life [43,44,45].

The lowest oxygen concentration to delay or prevent CI symptoms was 1 kPa (Figure 7). A lower oxygen level (0.5 kPa) resulted in necrosis and fungal infection (data not shown), probably because of excessive fermentation. It was reported that MG ‘Bermuda’ tomatoes stored at 22 °C under 0.5 kPa O_2_ developed identical symptoms after three days of storage [35].

### 4.2. Low oxygen Storage Alleviated CI in Tomato Which Might Be Related to Lower Oxygen Uptake and Improved Lycopene Synthesis

Tomatoes cultivated with FR during cultivation and kept at 1 kPa O_2_ during cold storage were shown to completely alleviate CI symptoms (Figure 5B) in MG fruit. This confirmed our previous findings that FR addition during cultivation suppressed CI incidence [29]. It was observed that MG tomatoes cultivated with FR initiated colour development at higher firmness [29]. It means that tomato cultivated with FR, although they had the same firmness as those cultivated without FR at harvest, maintained higher firmness during cold storage (Figure 6). Excessive firmness loss during cold storage and during shelf-life is often regarded as one of the main symptoms of CI in tomato with firmness retention associated with lower decay and higher membrane integrity [46,47]. Improved cold tolerance of FR cultivated tomatoes might also be attributed to thicker cuticle wax layers [48] which in turn might lower the oxygen consumption rate (Figure 8). On contrary, no significant difference was on weight loss (Figure S3). This might be attributed to comparably high relative humidity during the shelf-life (>95% RH) which suppress weight loos induced-transpiration from the fruit [49].

Our findings suggests that when low oxygen storage is applied to accompany long cold storage or transport, higher CI tolerance will result in shelf-life extension when tomatoes are grown with FR in greenhouses or grown in the field characterised by a low red to far-red ratio.

## 5. Conclusions

This study assessed the application of low oxygen either alone or in combination with far-red cultivated tomatoes on CI development. Results obtained showed the efficacy of low oxygen in minimising CI in tomato. CI tolerance is improved when low oxygen storage of MG tomatoes is combined with FR lighting during cultivation, especially when stored at 2 °C. This is likely due to lower oxygen uptake that allowed for to uninterrupted lycopene production and less softening during shelf-life for prior cold stored MG tomatoes kept at 1 kPa O_2_ and 0 kPa CO_2_.

## Data Availability

The raw data will be made available upon request.

## Supplementary Materials

The following are available online at https://www.mdpi.com/article/10.3390/foods10081699/s1, Figure S1: Spectra of ‘PhysioSpec’ Greenhouse white LED light and far-red light and the spectra of the ‘PhysioSpec’ Greenhouse lamp with only white LED light; Figure S2: Weight loss percentage of MG tomatoes during cold storage at 2 °C accompanied by 1 kPa, 5 kPa or 21 kPa O_2_ (low oxygen control) and 21 kPa O_2_ at 12 °C (temperature control), and subsequent shelf-life at 20 °C; Figure S3: H_2_O_2_ levels (nmol g^−1^ FW^−1^) of MG tomatoes during cold storage at 2 °C accompanied by 1 kPa, 5 kPa or 21 kPa O_2_ (low oxygen control) and 21 kPa O_2_ at 12 °C (temperature control), and subsequent shelf-life at 20 °C.
