# Supplementary figures and images for: Low Oxygen Storage Improves Tomato Postharvest Cold Tolerance, Especially for Tomatoes Cultivated with Far-Red LED Light
Source: Foods. 2021 Jul 22;10(8):1699. doi: 10.3390/foods10081699 (PMC8391604; doi:10.3390/foods10081699)

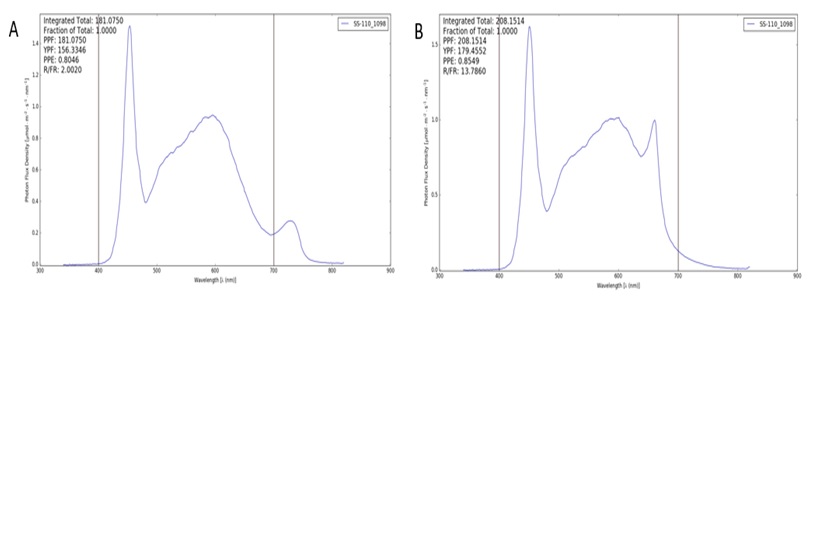

Supplement: Supplementary file 1 [file foods-10-01699-s001.zip › foods-1279684-SI/Fig S1.jpg]

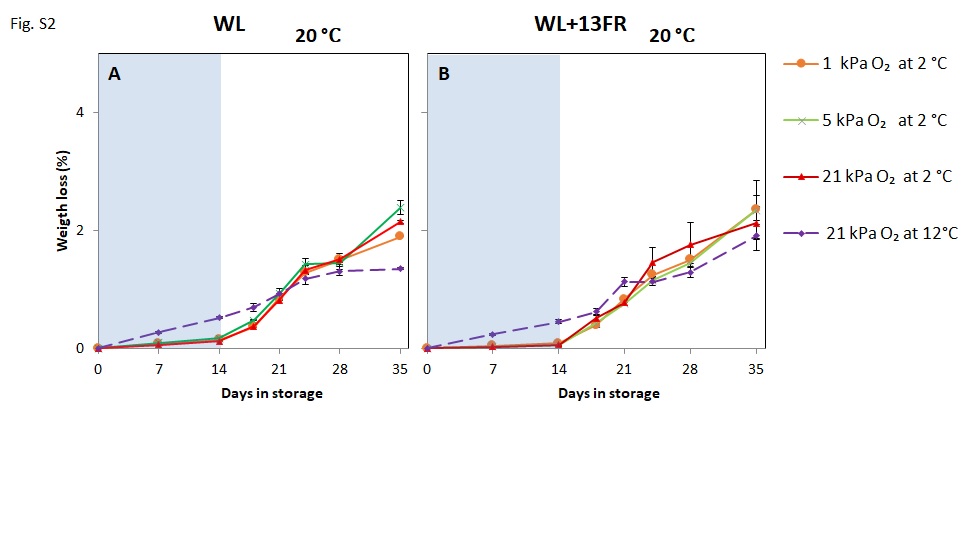

Supplement: Supplementary file 1 [file foods-10-01699-s001.zip › foods-1279684-SI/Fig S2.jpg]

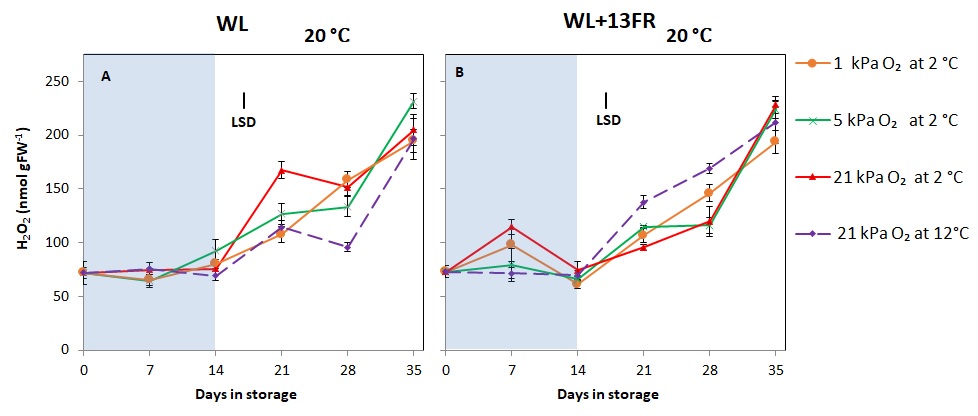

Supplement: Supplementary file 1 [file foods-10-01699-s001.zip › foods-1279684-SI/Fig. S3.jpg]
